# Supplementary material for: Rural-urban differentials in the prevalence of diarrhoea among older adults in India: Evidence from Longitudinal Ageing Study in India, 2017–18
Source: PLoS One. 2022 Mar 16;17(3):e0265040. doi: 10.1371/journal.pone.0265040 (PMC8926275; doi:10.1371/journal.pone.0265040)
Supplement: Attachment — Submitted filename: Response to Reviewers.docx [file pone.0265040.s001.docx]

Dear authors,

I would like to ask you to read the reviewers' comments and suggestions carefully. The reviewers still find so many shortcomings in the paper. I suggest to revise the paper and resubmit it. The revised version could be sent to new reviewers.

**Reviewer #1:** Although the authors have done few changes in the manuscript, most of the parts still need serious modifications. Authors should consider the following comments as constructive that will help them to make the manuscript more suitable for publication. Sentences in the abstract has not been reframed. Authors are recommended to read all the sentences. English should be checked properly as there are noticeable problems with prepositions, verbs. Sudden use of words like “Moreover”, “While” and “however” throughout the paper should be avoided.

**Response: The authors agree with the comment. The changes are now incorporated in the manuscript.**

Sentences like “Diarrhoeal diseases are seen among all age group” is not at all recommended.

**Response: The sentence is now reframed.**

Authors have not rewritten the abstract as recommended in the last review comments.

**Response: Dear reviewer, the entire abstract is now changed.**

Under Methods section “during 2016-2017” should be moved to any other sentence as it is not having any meaning in the present sentence. It seems authors have only taken into consideration the specific comments to be changed in the abstract. Policy recommendations are still very broad in the abstract part.

**Response: The sentence is now reframed. Dear reviewer, the study found that diarrhoea among older adults is significantly more prevalent in rural areas than in urban areas. Therefore, authors recommend the policy makers to focus more on rural areas to reduce the overall residential gap specifically and in general to reduce the overall burden of diarrhoea among older adults.**

“About 15 per cent of older adults in India were suffered from diarrhoea” reframe with correct forms of verbs.

**Response: The sentence is now reframed with correct form of verbs.**

Discussion:
“This study shows that the prevalence of diarrhoea is 7.7 percentage points higher in rural areas than urban areas”- avoid repeating the percentage from the results in the discussion part. But authors should discuss about the results and the main crux.

**Response: The sentence is reframed.**

“However, the finding is not similar with previous research in India [44, 51].” Should be reframed.

**Response: We have reframed the sentence.**

“Our study contradicts the existing literature and shows that the odds of older adults suffering from diarrhoea were higher among those who belonged to richer section of population [44, 53].
” Try to discuss this more as this is an important finding. Also check the analysis as the results are opposite in table 3.

**Response: Thank you for the comment. Given the dearth of literature that could possibly explain this unusual relationship, we will take up a deeper analysis of the economic status of the older adults and their odds of having diarrhoea in future.**

Paragraph starting with “Geographical differences in prevalence of diarrhoea” should be rewritten. There should be more discussion on the important findings from the study. Also, the discussion ends abruptly.

**Response: Thank you for the comment. We have re-written the paragraph.**

Strengths and limitations

“Therefore, very few studies have dealt with the older adults [57].” Not clear
This section should be written more clearly as the authors are suddenly starting to write about the limitations. Everything is getting mixed up.

**Response: The authors agree with the comment. The sentence is now removed. The strength and limitation section is reframed for better understanding.**

English needs to be checked throughout the manuscript. Errors in verbs, singular plural, prepositions can be found throughout the paper.

**Response: The paper is now edited by a native English speaker**.

References: References were not checked according to the last comment provided. Many references are not up to date. Check references 12, 10 and try to update them if necessary.
Kumar Panda Leuven SK, Kumar Bastia A. Anti-diarrheal activities of medicinal plants of Similipal Biosphere Re-serve, Potential Antibacterial Agent(s) against Foodborne Pathogens View project. Int J Med Aromat Plants. – wrong reference.

**Response: References are changed**

Many references are not having publication year and page numbers. There is no uniform style. In spite of giving comments in the first review about the references, the authors failed to check the references. Authors need to check each reference and write them properly. Avoid writing responses to comments as “changes incorporated” when authors have not done any changes in the reference section.

**Response: References has now been changed**

Tables
“May consider reordering of the variables. First may give soci0 demographic, then economic and household variables” This comment was given previously also. But the authors did not do the reordering of variables in all the tables. But in the response, they have written that they have made the changes. They should start with age, sex and then the other socio demographic, economic and household variables. Though the authors have stated this specific comment has been incorporated, the same is not the case. They have not modified the variables.

**Response: Dear reviewer, authors had arranged the table 4 as per your suggestion. Apologize for not making change in table 1 & 3. Now we have arranged the table 1 & 3 too as per your suggestion. Kindly refer to Table 1, 3 & 4.**

In table 3

Source of drinking water - adults suffering from diarrhea

Unimproved 10.9 %

Improved 15.2 %

This result is very shocking. Nothing has been mentioned about this result in the whole paper. On the other hand, the results are opposite in the table with logistics regression. Authors may check the analysis for both the tables. And then mention them in results and discussion.

**Response: Dear reviewer, authors are also shocked by this inconsistency. We have checked the analysis again and found the same results. The reason for the opposite results in the logistic table might be because of the adjusted results.**

Discussion person can discuss this issue in discussion section. Another important finding that “prevalence of diarrhoea was more among underweight older adults” has also not mentioned in the discussion section. Discussion section should be properly written with focus on the interesting findings from the study along with linking with the previous literature. Similarly, the issue of living in kutcha pakka house is also missing from the discussion. Authors should go through the tables, results section and then write the discussion.

**Response: We have tried to incorporate the important findings of the paper. However, due to dearth of literature on the topic, at times it is difficult to support it with literature.**

Reviewer #2: The authors have worked so nice. The paper sounds good. I recommend editor to ask the author for some minor revisions like:

1. Write the abstract in a comprehensive way. Not copy and paste from the manuscript.

**Response: Dear reviewer, the authors edited the abstract as per your suggestion.**

2. Outcome variable is something which really comes out from the analysis. Not from the data set. So, authors can rename the outcome variable or can constract or recode the outcome variable.

**Response: Dear reviewer, I agree with the comment. The outcome variable was assessed using the question “whether, in the past two years, the respondent was diagnosed with diarrhoea by a health professional?” The variable was coded as no and yes in the dataset.**
