# Supplementary material for: Rural-urban differentials in the prevalence of diarrhoea among older adults in India: Evidence from Longitudinal Ageing Study in India, 2017–18
Source: PLoS One. 2022 Mar 16;17(3):e0265040. doi: 10.1371/journal.pone.0265040 (PMC8926275; doi:10.1371/journal.pone.0265040)
Supplement: Attachment — Submitted filename: Response to Reviewers.docx [file pone.0265040.s002.docx]

Reviewer #1: Reviewer is still not convinced with few results coming from the tables. Also in spite of correcting the references, there are still modifications to be done as in few references the authors have provided the year in brackets after the name of the author and in some places they have provided the year without brackets after the journal name. Not sure what style they have followed.

**Response**: The authors have followed Vancouver style using Zotero. The references are now edited using the same software.

There are some other observations too

“Our study contradicts the existing literature and shows that the odds of older adults suffering from diarrhoea were higher among those who belonged to richer section of population [44, 53].

the results are opposite in table 3.

Also no argument has been provided in the discussion part. While explaining the results of table 3, authors also have missed to write about this result.
Inspite of pointing this in the last comments this has not been mentioned in the results section. Authors should mention everything coming from their study. Presentation of only selective results from table 3 is not recommended.

**Response:** Dear reviewer, relationship of diarrhoea with wealth quintile has been added in results section of table 3. Also we have added in the discussion section.

This is a very important finding. Source of drinking water - adults suffering from diarrhea.

Unimproved 10.9 %

Improved 15.2 %

This has yet not been written in the results section.

**Response:** This now added in the result section.

>The highest rural-urban difference in the prevalence of diarrhoea was observed among older adults who lived in kutcha houses. Studies conducted in Bangladesh and Ethiopia revealed the same findings [40–44].

References 40 to 44 includes India, Indonesia, along with Bangladesh and Ethiopia. They are not only on Bangladesh and Ethiopia.

Also ref 40 talks about "Among the individual food-hygiene variables, children who lived in the house with less dirty sewage had significantly lower diarrhea prevalence" and not directly on kutcha pakka houses. Authors can be more descriptive while citing references so that the sentences becomes self explanatory.

**Response:** Dear Reviewer, we apologize for the mistake. We have now added all the countries mentioned in reference 40-44. However, we have deleted India since it was on Risk of Adverse Pregnancy Outcomes among Women Practicing Poor Sanitation in Rural India: A Population-Based Prospective Cohort Study (Reference 44).

Dear Reviewer, we have rephrased the sentence.

>Also reference 43 by Luby did only talk about handwashing practices and diarrhoea among children. Why are authors citing references which are not talking about kutcha pakka houses. This is a wrong practice. Also ref 44 is on Risk of Adverse Pregnancy Outcomes among Women Practicing Poor Sanitation in Rural India: A Population-Based Prospective Cohort Study.

**Response:** Dear Reviewer, we apologize for the mistake. We have now specifically mentioned what each of the study deals with. Also, we have deleted reference 44 in this context.

>Findings from a previous study supported our results that older adults with high education had lower risk of diarrhoea [45]

Ref 45 is on Incidence and Correlates of Diarrhea, Fever, Malaria and Weight Loss Among Elderly and Non-Elderly

Internally Displaced Parents in Cibombo Cimuangi in the Eastern Kasai Province, Democratic Republic of the Congo. This talked about role of spouse's education. I am not sure how are the authors citing there references linking to their studies directly.

**Response:** Dear Reviewer, we have removed reference 45 and added “Incidence and Determinants of Acute Diarrhoea in Malaysia: A Population-based Study”.

>Logistic regression results reveal that the prevalence of diarrhoea was positively associated with higher age of older adults, who belonged to Scheduled Tribe (22 per cent higher risk) and OBC social group (24 per cent higher risk). This finding is consistent with a study carried out in India [46].
Ref 46 is on under 5 children in India. Authors should not directly link to them. Even if linking they should mention about the study done among under 5 children. The representation not proper.

**Response:** Dear Reviewer, we apologise for the mistake. We have made corrections accordingly and mentioned that the study was done among children under the age of five years.

>A higher concentration of diarrhoea was found in central and northeastern parts than in southern states of India. This could be because of unequal access to health care facilities, use of untreated drinking and low hygiene practices.

it will be better if the authors can find any literature supporting their argument.

**Response:** Dear Reviewer, though we were unable to support our study finding with literature on older adults, we could present some studies that dealt with children.

>Discussion part is still not adequate as this study has many important and striking findings.

>Moreover, the study reveals that older adults who belong to the Christian religion were more likely to have diarrhoeal risk than Hindu older adults. However, this finding is inconsistent with previous research in India [37,46].

**Response:** Dear Reviewer, we have tried to improve the discussion section.

In reference number 37 and 46- both the studies are on Children and also mentioned about Muslim children suffering more than Other religion. I am not sure whether authors can use these literature to show inconsistency, as their own results are concerned about the Christians and Hindus. and also on older adults.

**Response:** Dear Reviewer, we agree with you. However, due to lack of literature on diarrhea among older adults in India we could not support our finding. Hence, relied on literature on children in India.

So many mistakes and improper use of literature in Discussion part is unacceptable.

I suggest all the authors should go through the manuscript attentively and focus on their results and the discussion part. More literature review is required. They should also go through few other published papers and follow how to write the discussion part. They should resubmit the manuscript when they feel it is ready for publication.

**Response:** Dear Reviewer, we have tried to improve the discussion section.

Reviewer #2: If possible, please prepare the map of India propoerly by using ARC GIS software. Put lat-long and other spatial details in the map.

**Response:** Dear reviewer, the map is made using Arc GIS software. The spatial details are added.
